# The protective role of vitamin C in the management of COVID-19: A Review

**DOI:** 10.1186/s42506-021-00095-w

**Published:** 2021-12-11

**Authors:** Mohammad Sarowar Uddin, Md. Shalahuddin Millat, Prodip Kumar Baral, Mahmuda Ferdous, Md. Giash Uddin, Md. Shahid Sarwar, Mohammad Safiqul Islam

**Affiliations:** 1 Department of Pharmacy, Noakhali Science & Technology University, Noakhali, 3814 Bangladesh; 2 Department of Pharmacy, University of Chittagong, Chittagong, 4331 Bangladesh

**Keywords:** COVID-19, Vitamin C, Pathogenesis of COVID-19, Immunity

## Abstract

**Background:**

The outbreak of coronavirus infectious disease-2019 (COVID-19) is globally deemed a significant threat to human life. Researchers are searching for prevention strategies, mitigation interventions, and potential therapeutics that may reduce the infection’s severity. One such means that is highly being talked in online and in social media is vitamin C.

**Main text:**

Vitamin C is a robust antioxidant that boosts the immune system of the human body. It helps in normal neutrophil function, scavenging of oxidative species, regeneration of vitamin E, modulation of signaling pathways, activation of pro-inflammatory transcription factors, activation of the signaling cascade, regulation of inflammatory mediators, and phagocytosis and increases neutrophil motility to the site of infection. All of these immunological functions are required for the prevention of COVID-19 infection.

**Conclusion:**

Considering the role of vitamin C, it would be imperative to administrate vitamin C for the management of severe COVID-19. However, there is no specific clinical data available to confirm the use of vitamin C in the current pandemic.

## Background

Vitamin C or ascorbic acid or ascorbate is an inevitable cofactor for mediating countless enzymatic reactions that are accountable for numerous biological activities. It is deemed a robust antioxidant with strong anti-inflammatory and anti-microbial actions [1]. It possesses a wide range of biochemical and biological actions like antioxidant, phagocytosis, neutrophil chemotaxis, microbial clearance, and immunomodulatory, antiviral, and anti-inflammatory effects [2, 3] and improves natural killer cell and T cell proliferation [4]. Besides, vitamin C is required for the synthesis of nor-adrenaline [5], catecholamine [6], and adrenal steroids [7]. It also acts as a cofactor for peptidyl-glycine alpha-amidating monooxygenase that is needed for vasopressin’s endogenous synthesis [8]. Pre-operative administration of vitamin C reduces etomidate-induced adrenal suppression [9]. It has been extensively used to manage critically ill patients [10]. In addition, vitamin C enhances the immune system via several pathways, such as provoking the response of T lymphocytes, augmenting the activity of lymphocytes and phagocytes, increasing interferon levels [11], and scavenging reactive oxygen species (ROS) [12].

The severe acute respiratory syndrome coronavirus 2 (SARS-CoV-2) is now deemed as the global health burden [13]. The World Health Organization (WHO) announced COVID-19 as a global pandemic on 11 March 2020 [14]. As of 2 August 2021, over 199.02 million confirmed cases had been identified and more than 4.24 million people died of COVID-19 [15]. All ages people are affected by this virus, and elderly patients with comorbidities are getting severely ill, even death [14]. The symptoms are usually noticed between 2 and 14 days of coronavirus infection and include fever, cough, and shortness of breath [16, 17]. Acute respiratory distress syndrome (ARDS), septic shock, sepsis, heart failure, and viral pneumonia are the most common complications in severe cases [18]. Viral pneumonia with severe acute respiratory failure may lead to death [19]. Besides, multiple organ dysfunctions may provoke cytokine storms and uncontrolled acute inflammation [20, 21].

The Strategic Advisory Group of Experts (SAGE) on immunization of WHO has recommended several COVID vaccines like Astrazeneca, Sinopharm, Pfizer, Moderna, Covax, etc., to bring out the current pandemic under control [22]. It is found that people are getting infected even after taking the vaccine and various adverse effects are noticed following vaccination [23]. So, the questions arise about the safety and efficacy of COVID vaccines. In this hectic condition, social distancing; self-quarantine or home quarantine; hygiene practices such as mouth and nose covering during sneezing, coughing, and cooking; and sustaining a strong immune system are the best measures to impede the spread of COVID-19 infection [16, 24].

Despite there is no detailed clinical record for safe and effective antiviral drugs, some antiviral drugs that target the molecular pathways of COVID-19 have been used throughout the globe [25]. Among them, the only remdesivir has exhibited to be safe and effective in reducing the time to release of hospitalized COVID-19 patients [26]. The use of corticosteroids can only be considered for acute COVID-19 pneumonia patients for the short term to prevent disease advancement toward a severe form [27]. The extensively used chloroquine and hydroxychloroquine have revealed mixed outcomes in several studies and may be harmful owing to cardiac toxicity [28, 29]. So, we should adopt varying levels of protection against the COVID-19 pandemic. In this context, vitamin C may have positive outcomes on this acute viral infection. Moreover, vitamin C may alter susceptibility to respiratory tract infections by promoting coronavirus resistance [14, 30]. In fact, vitamin C-deficient people are prehensile to severe respiratory infections [3, 31], and supplementation with vitamin C may reduce this infection [31]. Therefore, the intervention to use vitamin C to treat COVID-19 infection is not worth surprising. However, in this review, we will outline the possible role of vitamin C supplementation to minimize COVID-19 pandemic complications.

## Epidemiological and clinical observations during COVID-19

Several clinical and epidemiological investigations outline the hypothesis regarding vitamin C status and its association with COVID-19. Some recent studies noted that COVID-19 is linked with the cytokine storm, pneumonia, ARDS, C-reactive protein (CRP), and heart failure [19, 32, 33]. In China, chronic fatality rates were 6–10% for individuals with cardiovascular disease, diabetes, hypertension, and chronic respiratory tract disease [34], whereas it is 1.4% in the USA [35]. Again, WHO reported that about 3.4% confirmed cases had died in COVID-19 as of March 3, 2021 (https://www.worldometers.info/coronavirus/coronavirus-death-rate).

**Fig. 1:**
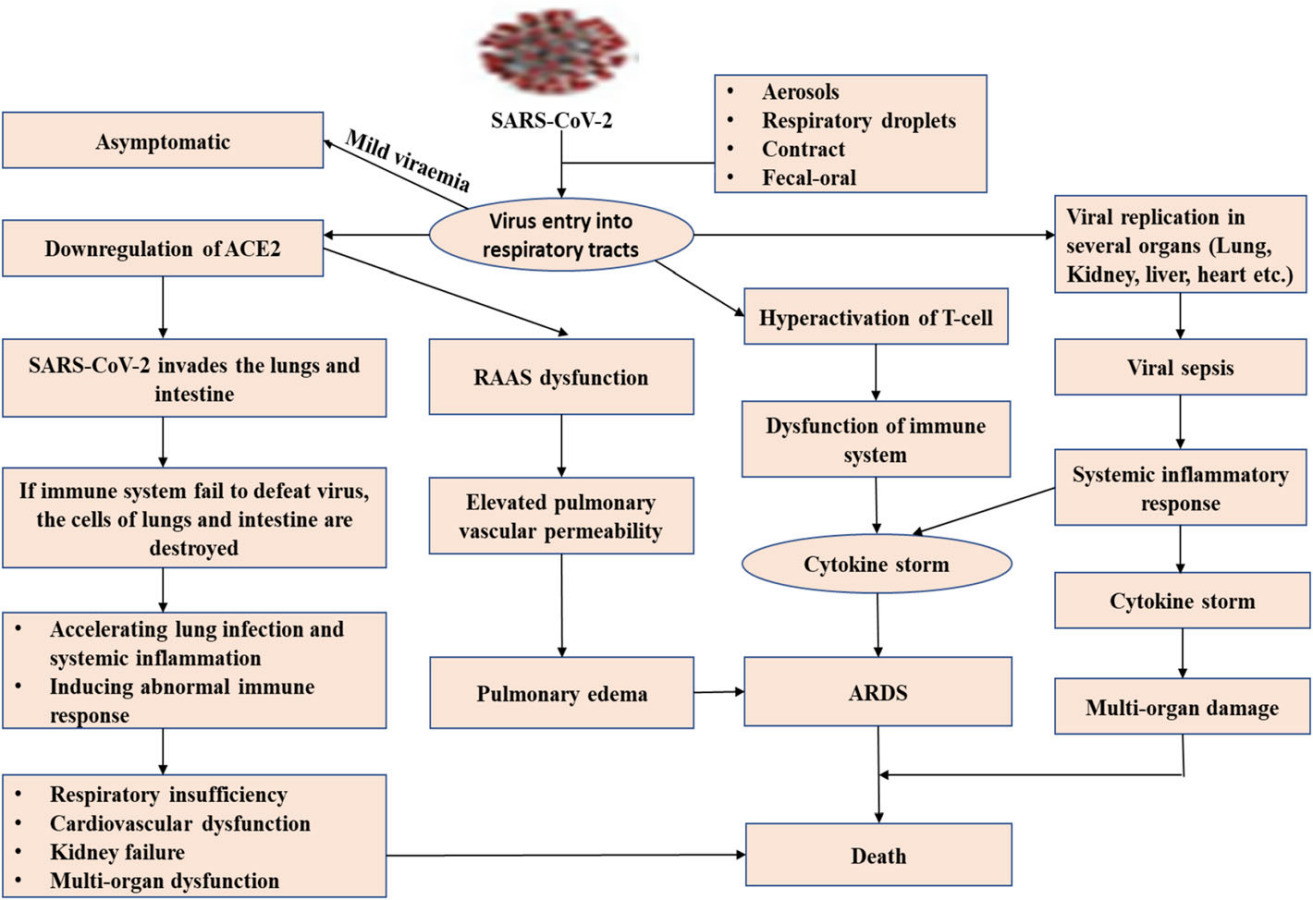
Hypothesized pathogenesis of SARS-CoV-2 infection SARS-CoV-2, severe acute respiratory syndrome coronavirus 2; ACE2, angiotensin-converting enzyme 2; ARDS, acute respiratory distress syndrome; RAAS, renin-angiotensin-aldosterone system

The SARS-CoV-2 virus is transmitted mainly via respiratory droplet, aerosols, contact, and fecal-oral [36]. A receptor-binding motif (RBM) of the inhaled virus binds to angiotensin-converting enzyme 2 (ACE2) of epithelial cells of the nasal cavity. ACE2 is the predominant receptor for SARS-CoV-2 binding. The aftermath of RMB and ACE2 binding is replication and propagation of coronavirus that ultimately lead to infected organ infection [37]. ACE2 is highly expressed in the bronchus, nasal mucosa, esophagus, lung, heart, kidney, bladder, stomach, and ileum, vulnerable to SARS-CoV-2 [38]. Viral replication is assumed to occur in the upper and lower respiratory tract’s mucosal epithelium following multiplication in gastrointestinal mucosa and yield to a mild viremia [39]. Older people are at high risk owing to their diminished immune response and decreased ability to regenerate the damaged epithelium [36]. Few patients remain asymptomatic through controlling the infection at this point. Some patients may develop non-respiratory symptoms, including diarrhea, kidney failure, heart, and liver injury, indicating multiple organ dysfunction [33, 40]. The hypothesized pathogenesis of COVID-19 infection is graphed in Fig. 1.

## The topicality of vitamin C to the COVID-19 pandemic

Vitamin C is deemed to have conducive effects in critically ill patients. It is a free radical scavenger that influences cellular immunity, anti-inflammatory properties, and vascular integrity, and acts as a cofactor in producing endogenous catecholamines [41]. COVID-19 causes life-denunciatory respiratory diseases in humans like severe acute respiratory syndrome (SARS) and the Middle East respiratory syndrome (MERS) [42]. The virulence mechanisms underlying COVID-19 outcomes are not fully understood yet [43]. Different cellular mechanisms such as dipeptidyl peptidase-4 receptor (DPP-4) binding, retinoic acid-inducible gene-I-like receptors (RIG-I) and melanoma differentiation-associated 5 (MDA5) host-recognition, papain-like protease (PL-pro)-mediated replication, and breakdown of M-protein-mediated type-1 IFN (Interferon) initiation evasion have been identified in the closely linked COVID-MERS virus [44]. Of them, DPP-4 has been shown to closely link with COVID-19 in the case of the S1 domain of the spike glycoprotein, pointing out that it would be a primary intensive factor of COVID-19 infections [45]. During COVID-19, an expansive and unrestrained release of pro-inflammatory cytokines or cytokine storm occurs that is also observed in MERS and SARS-CoV-1 [18]. Clinically, this cytokine storm induces systemic inflammation and respiratory inflammation and multiple organ failure [20]. Vitamin C supplementation has shown conducive effects in infections and sepsis. As severe COVID-19 may induce acute respiratory distress syndrome (ARDS) and sepsis, high doses of vitamin C supplementation may contribute to ameliorating inflammation in patients with COVID-19. In fact, vitamin C-deficient individuals are highly susceptible to systemic inflammation, and severe respiratory infections observed throughout the course of COVID-19 [31].

## Evidence of antiviral action of vitamin C

Vitamin C supplementation has demonstrated a wide range of antiviral effects against several types of viral infections [46]. Decreased levels of vitamin C have been observed in different viral infections [13] such as sepsis, sepsis-related acute respiratory disease syndrome (ARDS) [47], and other critical conditions of illness [3]. In viral infections, vitamin C is crucial for destroying neutrophil [48] accumulated within macrophages and is accountable for T cell maturation [49]. This enhances phagocytosis and apoptosis [3]. In several murine models, vitamin C has promoted survival from lethal infection [46]. Treatment with vitamin C (50 mg/kg) in the Venezuelan encephalitis virus-affected mice revealed 50% mortality of the controls with less nitric oxide (NO) content and lipid peroxidation products. The treatment group exhibits an increased survival rate (50%) in comparison with untreated infected mice (0%) [50]. A previous study noted that mice infected with the influenza virus could not yield vitamin C, and mice not receiving vitamin C supplementation showed higher lung pathology scores [51].

Vitamin C decreased mortality rate in dose-dependent fashion (100%, 80%, and 50% at 0, 125, and 250 mg/kg/day) in H1N1 (hemagglutinin type 1 and neuraminidase type 1) viral-induced pneumonia [52]. Mice infected with the rabies virus demonstrated nearly 50% mortality while treating intramuscular 100 mg/kg vitamin C daily in comparison with untreated infected mice [46]. Vitamin C supplementation (300 mg/day) in influenza-induced pneumonia is protected from severe infection and reduced the duration of hospital stays [46]. Another study in 133 patients demonstrated that the administration of vitamin C promotes the protection (odds ratio 0.25) from herpes simplex keratitis [53]. Some current studies reported vitamin C as a potential intervention against the coronavirus [16, 54]. Hence, it is hypothesized that vitamin C supplementation may reduce the severity of the current COVID-19 pandemic.

## Immunomodulatory role of vitamin C

**Fig. 2:**
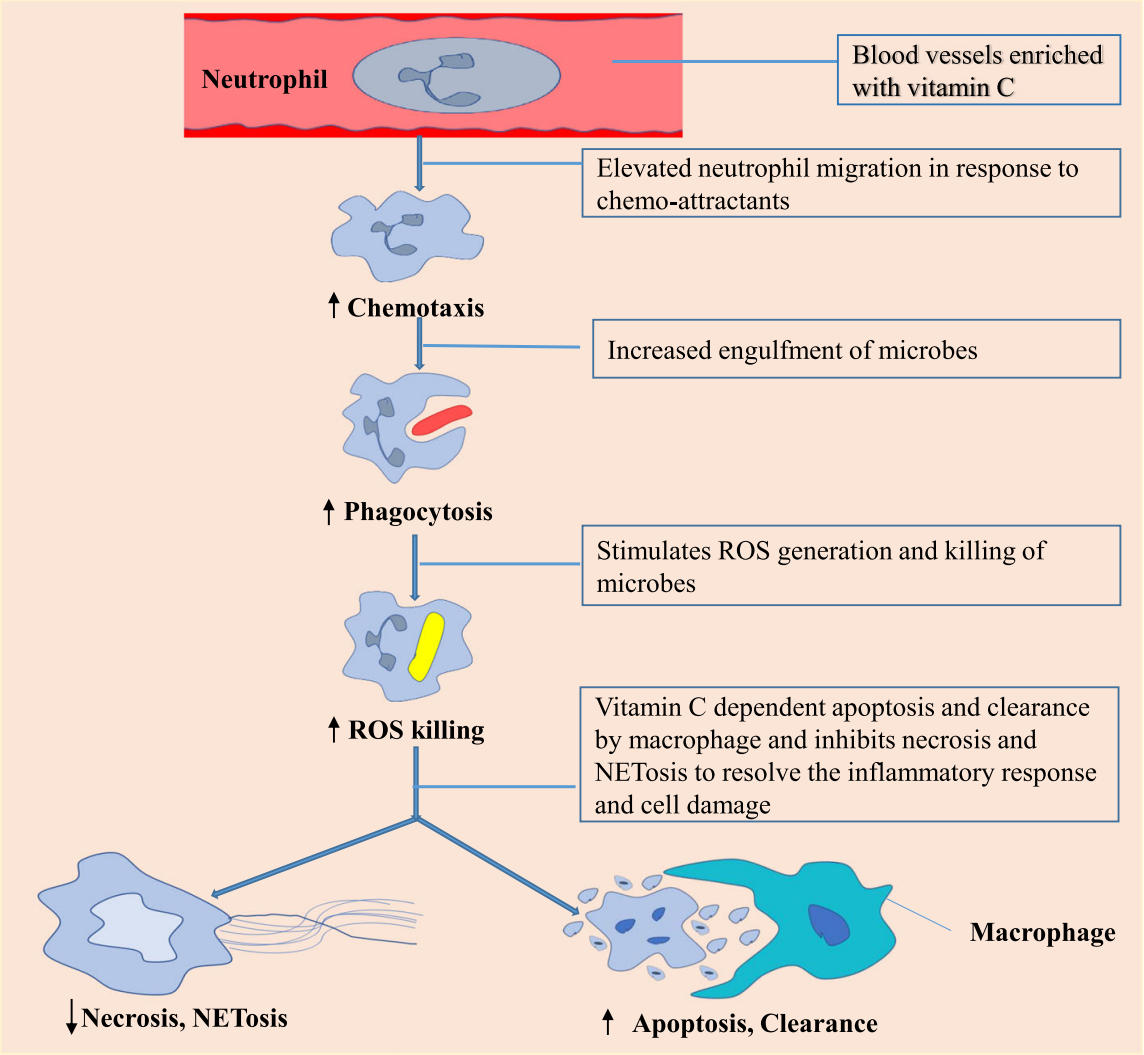
The role of vitamin C in immune cell functions

The immune system is a complex defense mechanism comprising innate and adaptive responses [31]. The innate immune system recognizes and destroys “non-self” rebuffs via inflammatory processes to repair the damage [55]. Vitamin C influences various pathways of immunity such as promoting epithelial barrier activity, migrating white blood cell (WBC) to infected sites (Fig. 2), controlling growth and function of innate and adaptive immune cells, killing microbes via phagocytosis (Fig. 2), and producing antibody [3, 31]. There is evidence that vitamin C may promote pneumonia patients’ health status [56, 57]. Previous findings reported that mice deficient in vitamin C, when infected with H3N2 (hemagglutinin type 3 and neuraminidase type 2) influenza, displayed worse results due to reduce IFN-a/b and increase IL (interleukin)-1a, IL-s1B, and tissue necrosis factor-a (TNF-a) levels [58]. When mice received vitamin C supplementation, these cytokine storm expression profiles were removed. A high dose of IV vitamin C (7.5–50 g) in acute Epstein-Barr virus infection (EBV) exhibited decrease EBV-IgG (immunoglobulin G) profiles, while EBV-IgM antibody profiles were negatively correlated with elevating plasma ascorbate concentration [59].

Patients with human T-cell leukemia virus, type 1 (HTLV-1)-associated myelopathy (HAM) or tropical spastic paraparesis were treated with oral vitamin C (35–40 mg/kg) for 3–5 days and exerted no changes in HTLV-1 antibody titer in serum and cerebrospinal fluid, revealing an immunomodulated effect [60]. A study reported that rabies vaccination with supplementation of oral vitamin C (2g) elevated serum IFN-a profiles, indicating that vitamin C stimulates interferon production [61]. Another study revealed that a diet containing vitamin C in a mouse model increased interferon production (62–145%) based on the inoculation of viral titer [62]. Vitamin C exhibits immunomodulatory effect by promoting interferon synthesis via signal transducers and activation of transcription 3 (STAT3) phosphorylation [58], enhancing survival in lethal infections [50], controlling cytokine storm-induced organ damage [51], and recycling oxidized quercetin and promoting its antiviral functions [63]. These immunological alterations suggest that vitamin C possesses a good immunomodulatory effect, and we should prioritize it against the current COVID-19 pandemic for pharmacological intervention.

## How does vitamin C work against infections as well as COVID-19?

The key reason for severe lung injury in COVID-19 patients is the oxidative stress and excessive free radicals generated from the dysfunctional immune system to kill the virus but end up wounding the patient instead. Vitamin C can reduce oxidative damage and neutralize these free radicals in the lungs. When there is an imbalance between oxidants and antioxidants, body organs’ damage occurs and progresses patients to severe disease [64]. We can improve the antioxidant status by administering sufficient vitamin C. The white blood cells (immune cells) are highly enriched with vitamin C, implying these immune cells’ functional roles. Vitamin C influences phagocytes’ functions, replicating viruses, production of interferon, and maturation of T-lymphocytes. Vitamin C also acts as a safeguard of these immune cells against oxidative damage when they clear out viruses from the body. Based on these knowledge, many hospitals in China, New York, and Shanghai have already started administrating intravenous vitamin C to manage the COVID-19 infections. However, anecdotal evidence of positive response from these hospitals suggests promising vitamin C results against COVID-19 [64].

## Clinical efficacy of vitamin C in critically ill patients without COVID-19

More than hundreds of animal studies have demonstrated that a few grams of vitamin C’s daily dose may prevent infections [65]. A pilot study on 24 critically ill patients with sepsis revealed that intravenous (IV) vitamin C administration reduces the Sequential Organ Failure Assessment (SOFA) scores and levels of pro-inflammatory markers (Table 1) significantly over the 4-day study period in patients who received 50 and 200 mg/kg per day vitamin C in comparison with patients who received placebo [66]. Interestingly, a randomized control trial did not change levels of inflammatory markers or SOFA scores in sepsis-induced ARDS patients (*n* = 167) after IV vitamin C 200 mg/kg per day administration for 4 days. However, the hospital stays and the mortality rate decreased significantly in the treatment group in 28 days (*p* = 0.03) [47]. Several controlled studies point out that the combination therapy of vitamin C, hydrocortisone, and thiamine had auspicious effects in sepsis or severe pneumonia patients [53, 67]. Again, a randomized control trial study in patients with septic shock compared the outcomes of the combination therapy of vitamin C (6 g per day), hydrocortisone (200 mg per day), and thiamine (400 mg per day) to hydrocortisone alone. The study’s findings showed that combination therapy did not affect the duration of a shock though significantly improved the SOFA score in treatment groups (*p* = 0.03) [68]. These clinical findings suggested that vitamin C may play a significant role in the management of COVID-19 infection.

**Table 1:** Role of vitamin C in the modulation of several complications [69]

Complications	Role of vitamin C
Cytokine storms	A large dose of vitamin C could prevent and manage cytokine storms
Inflammatory response syndrome	Vitamin C assists in regulating inflammatory response to prevent immune cell damage
Oxidative stress	Prevention and management of oxidative stress through a large dose of vitamin C administration
Acute respiratory distress syndrome	Vitamin C elevates ACE2 regulation and modulates the renin-angiotensin system to restore proper lung function
Hyperactivation of the other immune system responses	Vitamin C helps the immune system to fight against infection
Sepsis	Intravenous vitamin C administration reduces the SOFA scores and levels of pro-inflammatory markers significantly

## Clinical data on vitamin C in critically ill patients with COVID-19

A series of clinical trials related to COVID-19 have been launched or announced from the very beginning of COVID-19 to evaluate the therapeutic benefit of vitamin C alone or in combination therapy with one or more substances (e.g., zinc, vitamin D, hydroxychloroquine, and azithromycin) [70]. Randomized controlled trials are currently registered in the National Institutes of Health Clinical Trials (NIHCT)/National Clinical Trial (NCT) to examine monotherapy for severe COVID-19 treatment (to treat, or prevent COVID-19 in combination with hydroxychloroquine and other supplements [70, 71]. The dosing of vitamin C varies widely in these trials, ranging from 250 to 500 mg orally to 24 g IV daily.

Researchers from China have noted that they have successfully treated the moderate to severe COVID-19 patients (*n* > 50) with large doses of IV vitamin C (10,000–20,000 mg/day). The findings are no incidence of death and a shorter mean hospital stay than untreated COVID-19 patients [13]. Besides, the Shanghai Medical Association has recently endorsed the use of high-dose vitamin C for the management and treatment of hospitalized patients with COVID-19 [72]. A recent review points out that higher doses of IV vitamin C may be required to deplete cytokine storms in ARDS [73]. High doses of vitamin C are deemed to be administered IV route as they are thinly tolerated orally. However, bowel tolerance for vitamin C is elevated in many patients with the severity of illness. So, some patients may tolerate oral doses up to 200 g/day [74]. However, it should be kept in mind that vitamin C is not yet a standard treatment for COVID-19 owing to a shortage of evidence.

## Conclusion

In summary, vitamin C possesses positive impacts on curing of infection and this may play a protective role in the current COVID-19 pandemic through boosting the immune system. As a robust antioxidant, vitamin C helps in normal neutrophil function, scavenging of oxidative species, regeneration of vitamin E, modulation of signaling pathways, activation of pro-inflammatory transcription factors, activation of the signaling cascade, nuclear factor κB (NFκB), regulation of inflammatory mediators, gene regulation, phagocytosis, and signaling pathways in T-cells and increases neutrophil motility to the site of infection. These functions are very crucial for the prevention and treatment of COVID-19 infection. So, to develop strong immunity against COVID-19 infection, a regular administration of vitamin C is required. In healthy individuals, 200 mg/day of vitamin C is required to obtain saturated blood levels. The requirement of vitamin C increases during infection, and 1–2 g/day is recommended in this condition. Ongoing randomized clinical trials (RCT) are expected to give more definitive evidences.

## Data Availability

Not applicable.

## Abbreviations

COVID-19: Coronavirus infectious disease-2019
ROS: Reactive oxygen species
SARS-CoV-2: Severe acute respiratory syndrome coronavirus 2
WHO: World Health Organization
ARDS: Acute respiratory distress syndrome
CRP: C-reactive protein
ACE2: Angiotensin-converting enzyme 2
RAAS: Renin-angiotensin-aldosterone system
SARS: Severe acute respiratory syndrome
MERS: Middle East respiratory syndrome
DPP-4: Dipeptidyl peptidase-4
RIG-I: Retinoic acid-inducible gene-I-like receptors
MDA5: Melanoma differentiation-associated 5
PL-pro: Papain-like protease
IFN: Interferon
H1N1: Hemagglutinin type 1 and neuraminidase type 1
WBC: White blood cell
HFF: Human foreskin fibroblast
ASC-2P: 2-Phospho-ascorbate
H3N2: Hemagglutinin type 3 and neuraminidase type 2
IL: Interleukin
TNF-a: Tissue necrosis factor-a
IV: Intravenous
EBV: Epstein-Barr virus
IgG: Immunoglobulin G
HAM: Human T-cell leukemia virus, type 1-associated myelopathy
HTLV-1: Human T-cell leukemia virus, type 1
STAT3: Signal transducers and activation of transcription 3
SOFA: Sequential Organ Failure Assessment
NIHCT/NCT: National Institutes of Health Clinical Trials
